# Application of endoscopic technique in completely occluded anastomosis with anastomotic separation after radical resection of colon cancer: a case report and literature review

**DOI:** 10.1186/s12893-021-01202-6

**Published:** 2021-04-20

**Authors:** Junnan Gu, Shenghe Deng, Yinghao Cao, Fuwei Mao, Hang Li, Huili Li, Jiliang Wang, Ke Wu, Kailin Cai

**Affiliations:** Department of Gastrointestinal Surgery, Union Hospital, Tongji Medical College, Huazhong University of Science and Technology, Wuhan, 430022 China

**Keywords:** Radical resection of colon cancer, Severe anastomotic stenosis, Completely occluded anastomosis, Anastomotic separation, Endoscopic technique, Self-expanding metal stent

## Abstract

**Background:**

Anastomosis-related complications are common after the radical resection of colon cancer. Among such complications, severe stenosis or completely occluded anastomosis (COA) are uncommon in clinical practice, and the separation of the anastomosis is even rarer. For such difficult problems as COA or anastomotic separation, clinicians tend to adopt surgical interventions, and few clinicians try to solve them through endoscopic operations.

**Case presentation:**

In this article, we present a case of endoscopic treatment of anastomotic closure and separation after radical resection for sigmoid carcinoma. After imaging examination and endoscopic evaluation, we found that the patient had a COA accompanied by a 3–4 cm anastomotic separation. With the aid of fluoroscopy, we attempted to use the titanium clip marker as a guide to perform an endoscopic incision and successfully achieved recanalization. We used a self-expanding covered metal stent to bridge the intestinal canal to resolve the anastomotic separation. Finally, the patient underwent ileostomy takedown, and the postoperative recovery was smooth. The follow-up evaluation results showed that the anastomotic stoma was unobstructed.

**Conclusions:**

We reported the successful application of endoscopic technique in a rare case of COA and separation after colon cancer surgery, which is worth exploring and verifying through more clinical studies in the future.

## Background

As a common complication after radical resection of colon cancer, anastomotic complications hinder the smooth recovery of patients and make it difficult for clinicians to achieve efficient diagnosis and treatment [1, 2]. Severe anastomotic stenosis or even COA after radical resection of colon cancer is relatively rare, but it is a severe inconvenience to patients, because it delays the healing of anastomotic stoma and affects the anatomical reconstruction and functional recovery of the digestive tract [3]. It is difficult for clinicians to deal with this complication. The severity of the symptoms varies from patient to patient. Patients with severe cases may show symptoms, such as pain. In mild cases, slight discomfort is felt, or COA is only found during examination. Considering the anatomical structure changes in the COA location, the unknown nature of the intestinal tissue level, and the condition of the blood supply and other difficulties, clinicians usually opt for surgical treatment. Few studies have explored the effect of endoscopic techniques to solve the complications of postoperative COA of colon cancer. The direction of endoscopic dissection and the choice of options for dilating the stenosis need to be determined in endoscopic operations. No clear definition of anastomotic separation is found after colorectal cancer surgery, because when the anastomosis is split, broken, or ruptured after surgery, the patient has obvious clinical symptoms and examination features and thus receives timely therapeutic intervention. However, the stable presence of anastomotic separation in clinic has been rarely reported in the literature.

We introduce a case involving COA diagnosis and treatment with anastomotic separation after radical resection of sigmoid carcinoma by endoscopic technology.

## Case presentation

**Fig. 1:**
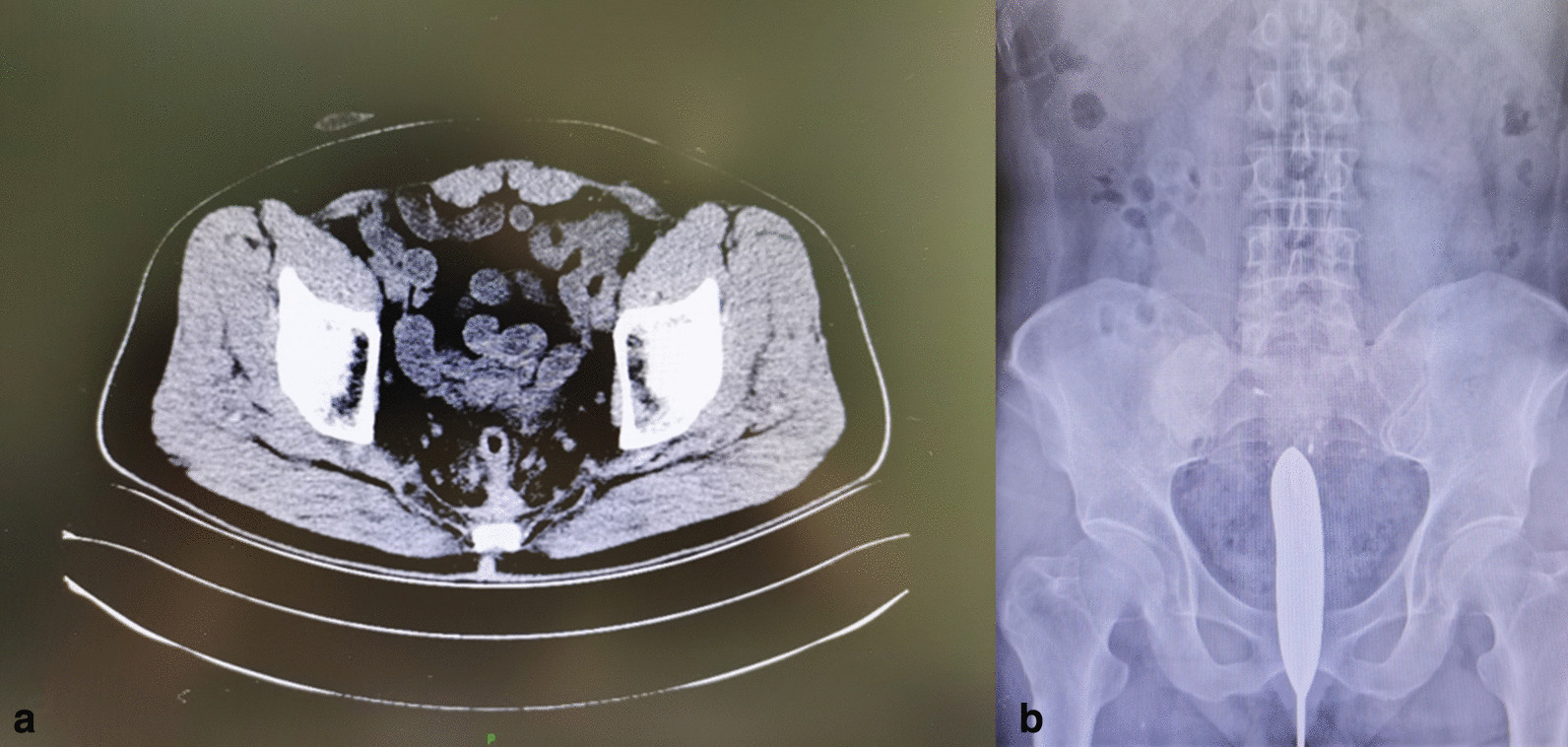
The imaging scanning of the patient. **a** Abdominal enhanced CT showed that the intestinal lumen above the anastomotic stoma was narrow and adhered to the anterior sacral fascia. **b** In the barium enema examination, the anastomotic stoma in rectum was completely closed and separated

A 58-year-old man underwent laparoscopic radical resection of sigmoid carcinoma and ileostomy in the local hospital in June 2017. After the operation, he underwent intermittent chemotherapy six times. His physical condition recovered, and the re-examination showed no tumor recurrence. The patient was expected to undergo ileostomy takedown in the local hospital in June 2018 and April 2019, respectively, but the operation was not performed because of the COA, which was found by colonoscopy. The patient was admitted to the Department of Gastroenterology, Union Hospital, Tongji Medical College, Huazhong University of Science and Technology, Wuhan, Hubei Province, China in May 2019. To address the difficult and complicated post-surgery problems of this cancer patient, the medical team conducted a comprehensive and careful related examination and disease evaluation. The abdominal enhanced computed tomography (CT) showed postoperative changes of sigmoid carcinoma. The intestinal lumen above the anastomotic stoma was narrow and adhered to the anterior sacral fascia. The stoma was unobstructed after the right lower abdominal fistulation (Fig. 1a). Further barium enema examination suggested that the anastomotic stoma in rectum is completely closed and separated (Fig. 1b). Blind ends were present on both sides of the sigmoid colon and the rectum, and the bilateral blind ends were approximately 3.0 cm apart. The laboratory examination of the patients showed that there was no obvious abnormality in the general hemogram and no significant change in the level of tumor markers. Physical examination showed no obvious discomfort.

Despite the presence of COA with anastomotic separation, the examination results indicated that the blind end of the anastomotic site grew well. No obvious defect was found in the surrounding tissue. At the same time, the additional surgical operation would bring more trauma. Therefore, after speaking with the patient and his family, we planned to perform endoscopic operation on the patient to treat his disease. If endoscopic surgery cannot solve the problem, then surgery can be performed immediately or postponed as an alternative.

**Fig. 2:**
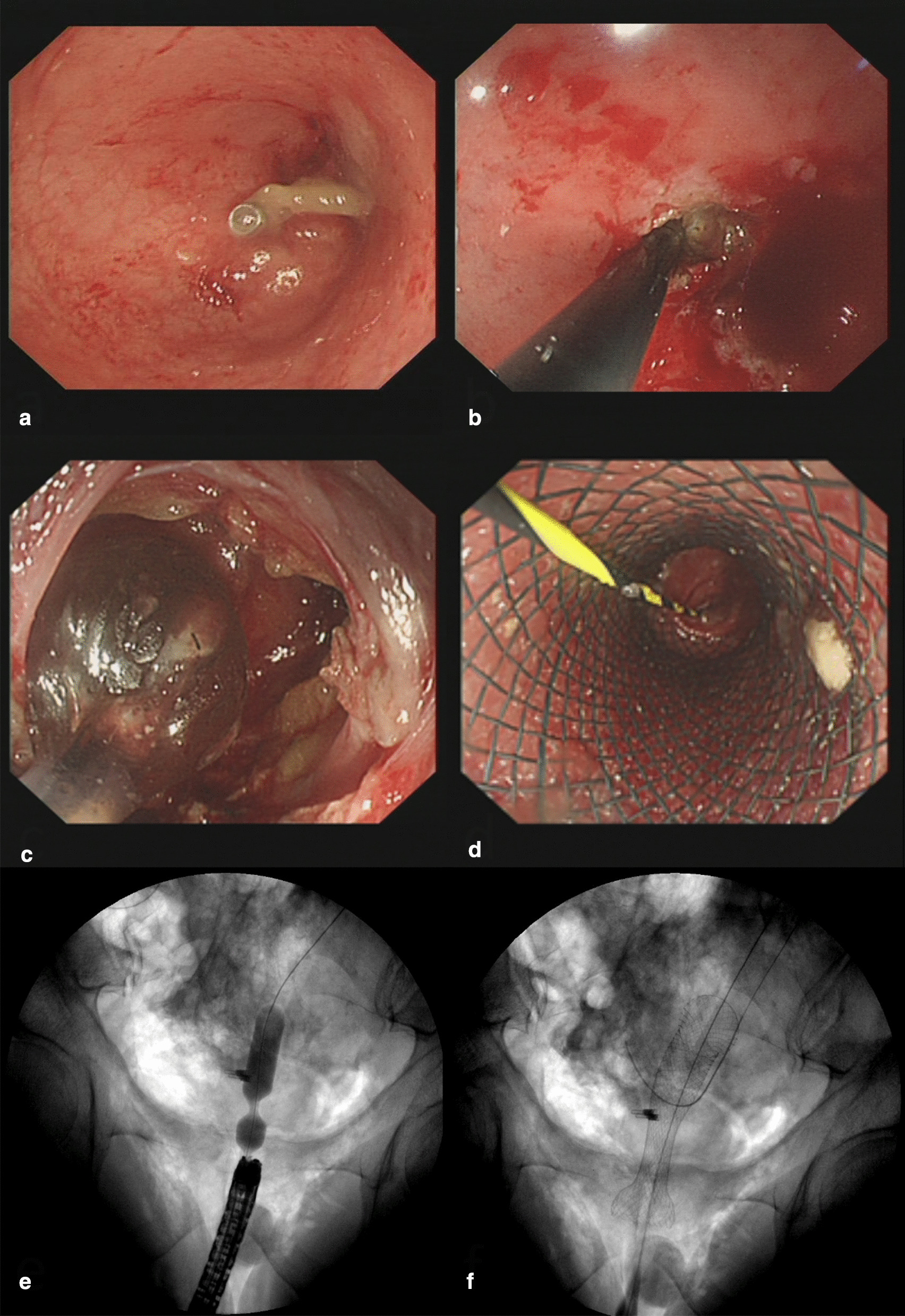
The patient's first endoscopic surgery procedure. **a** Titanium clips were labeled at the blind end of the proximal intestine. **b** The center of the blind end of the intestine was cut layer by layer with a hook knife. **c** Endoscopic application of dilating tubes and dilating balloons was performed to dilate the incised blind end of the intestinal canal. **d** The 60 mm * 20 mm self-expanding covered stent was inserted over the guide wire to bridge the two sides of the intestine. **e** On X-ray fluoroscopy, the dilated balloon was observed to gradually dilate the narrow blind end of the intestine. **f** Smoothly placed SEMS for dilation and bridging of the intestinal tube was visible under X-ray fluoroscopy

After the improvement of the preoperative examination, along with adequate preoperative communication and informed consent of patients, endoscopic surgery was proposed. In endoscopic surgery (Fig. 2), we first observed the two blind ends of the anastomotic stoma through colonoscopy. On the one hand, the COA can be seen at about 8 cm through the anus side of colonoscopy. The closure line showed a fine line scar, and the intestinal wall at the blind end was soft. On the other hand, the colonoscope entered through the ileostomy side to reach the rectal segment. The front of the blind end of the bowel was found to also be truncated and closed with a white linear scar, and a titanium clip was placed at the proximal blind end. The intestinal wall of this blind end was also soft when touched. Second, the distance between the blind ends of both sides of the intestine was 3–4 cm, as measured by fluoroscopy at different positions. Subsequently, the colonoscope re-entered through the anal side. The hook knife (KDL-620LR; Olympus Optical) was used to slowly cut through the scar tissue layer by layer in the center of the blind end of the intestinal canal, and the yellow adipose tissue became visible. The direction of incision was determined to be parallel to the direction of the titanium clip at the proximal blind end, as shown by repeated fluoroscopic observation. The adipose tissue was carefully cut open, and the proximal intestinal muscular layer was still not visible after 4 cm of advance. Third, EUS-FNA (19 Ga, 1.10 mm; Boston Scientific) puncture needle was used to puncture along the direction of the titanium clip under fluoroscopy, and a small amount of contrast agent was injected to observe the flow direction and the visualization of the intestinal canal. The intestine was visualized after the needle touched the titanium clip, and a yellow zebra guide wire was placed after the needle entered the proximal intestine. Finally, colonoscopy was performed through one side of ileostomy to confirm that the guide wire was located in the proximal intestine tube. The dilation tube and balloon (M00558430; Boston Scientific) were inserted over the guide wire, and the incised bowel was gradually dilated to 12 mm under fluoroscopy. Subsequently, a 60 mm * 20 mm self-expandable full-coated metal stent (Cook Medical, Bloomington, IN, USA) was placed over the guide wire to bridge both sides of the intestine, thereby achieving the repair of anastomotic closure with separation through the endoscopic operation. After completion of the operation, the patency of the stent was checked, no bleeding was found on the wound surface, and the anal canal was retained. Compared with the “drastic” surgical operation, this endoscopic surgery solved the intractable problem of COA with anastomotic separation with a more delicate and subtle operation and with minimal trauma. Endoscopic stent removal was performed on the patient 10 days later. The continuity of the bowel was observed intraoperatively, and an anal tube was placed in the proximal part of the anastomosis to observe the postoperative recovery.

**Fig. 3:**
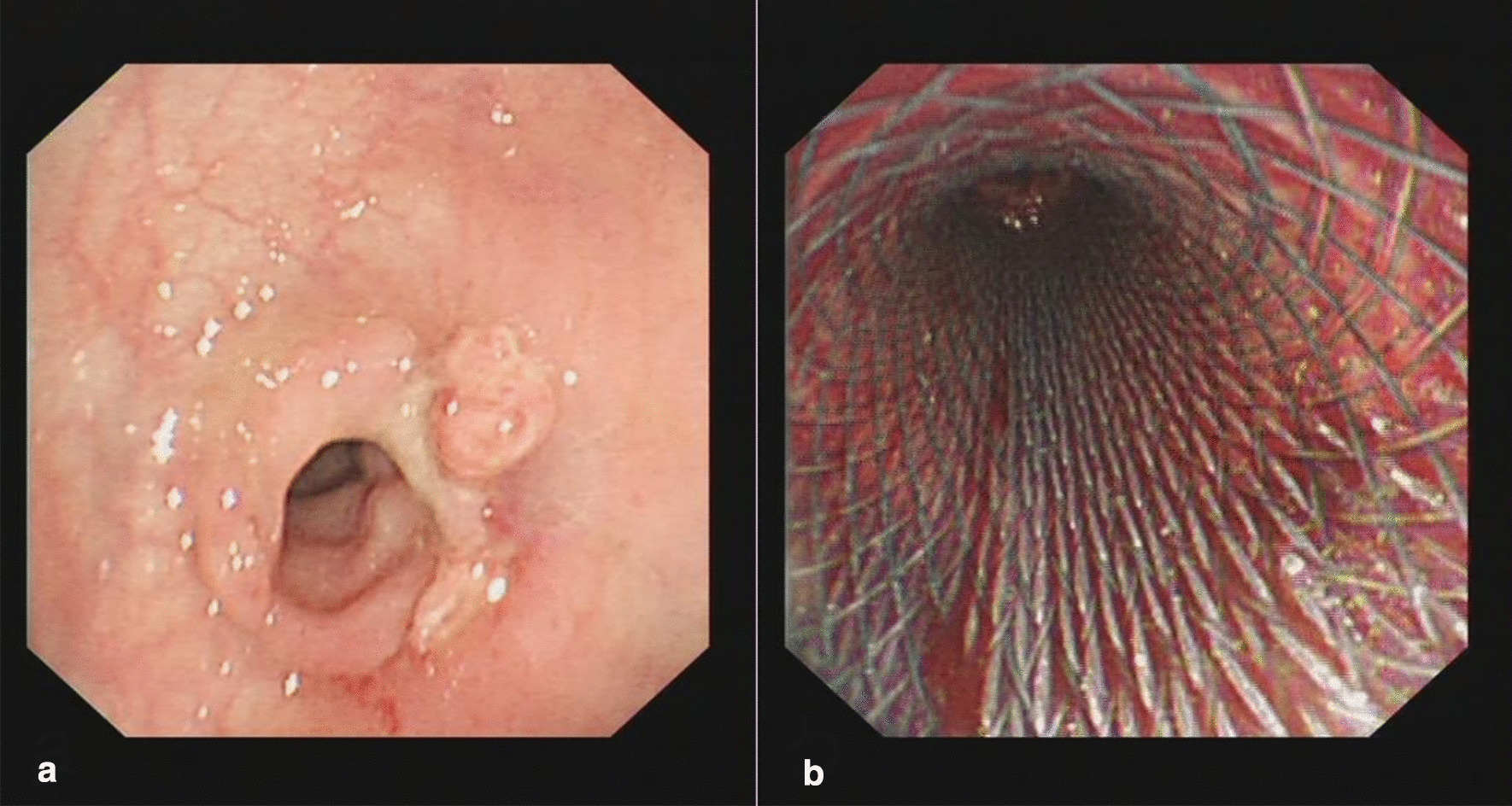
The patient's second endoscopic surgery procedure. **a** The original endoscopic recanalization site had stenosis with peripheral scarring hyperplasia. **b** The anastomotic stenosis was solved by endoscopic placement of a self-expending metal stent

One month later, stenosis was observed at the original anastomotic recanalization site during endoscopic examination, and scar-like hyperplasia was observed in the surrounding tissues. We successfully leaped over the narrow segment through inserting a new self-expandable full-coated metal stent of the same specification, and the stent expanded smoothly (Fig. 3). After 1 week, the patient underwent stent removal and came to our hospital for follow-up and reexamination for several times after discharge. The anastomotic stenosis of the patient was gradually relieved. At 1 year after the endoscopic recanalization procedure for COA with anastomotic separation, the patient came to our hospital in June 2020 for ileostomy takedown. No related complications occurred during and after the operation. No obvious abnormality was found in the relevant post-operation laboratory and imaging examinations.

**Fig. 4:**
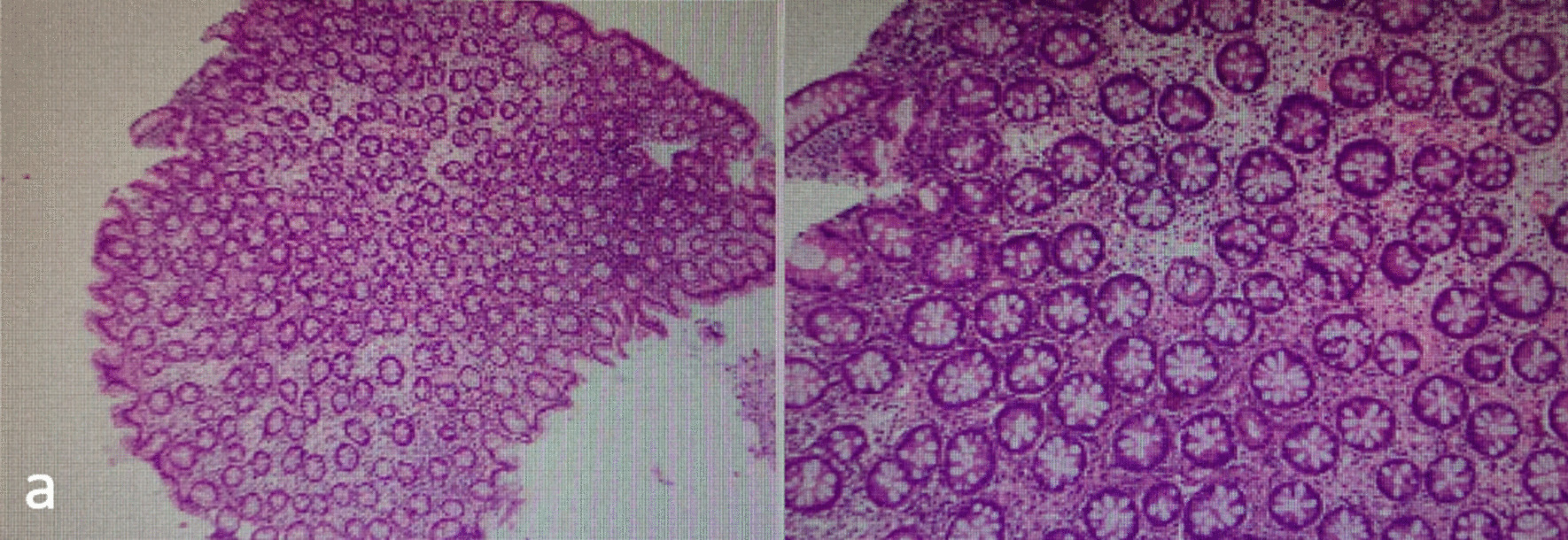
The pathological findings of rectal biopsy tissue. **a** The normal mucosal tissue was grown at the original endoscopic recanalization site

**Fig. 5:**
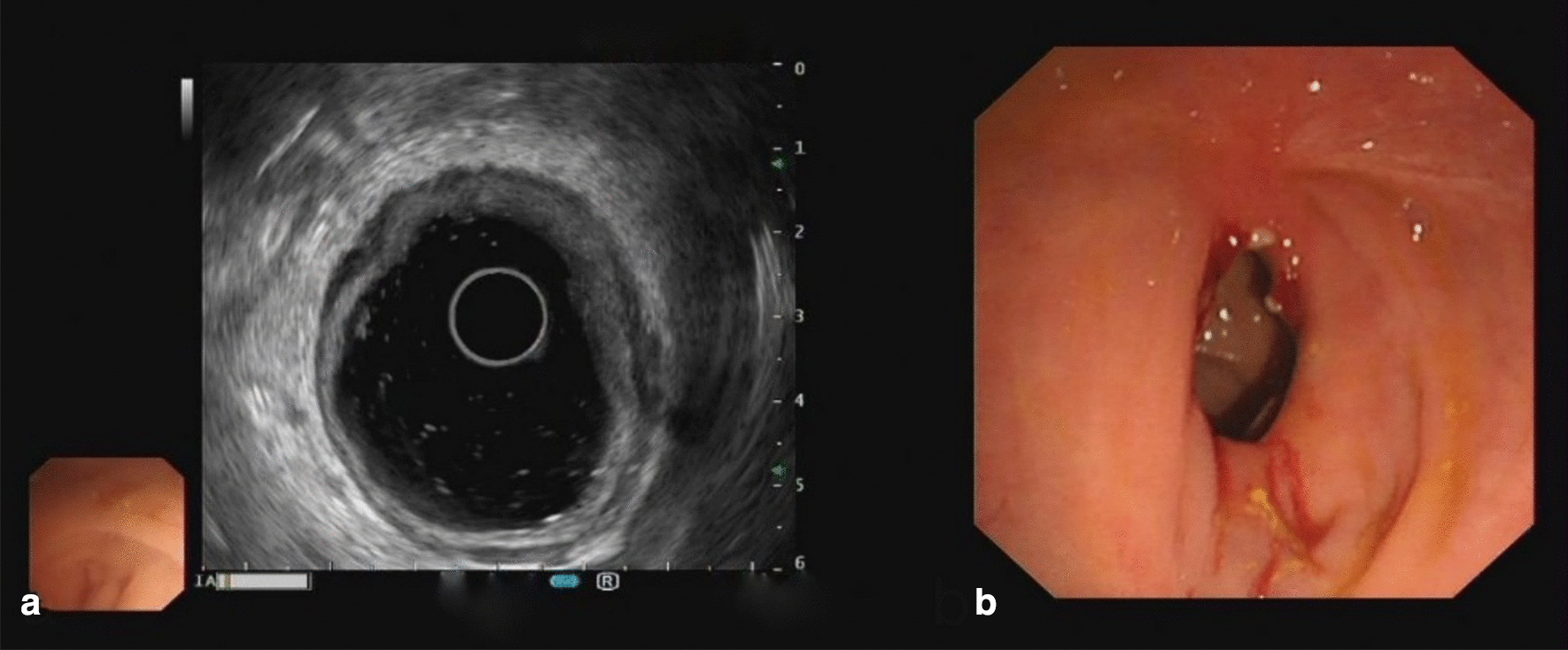
Endoscopic ultrasonography indicated a good prognosis. **a** The stratification of normal intestinal mucosal tissues at the original endoscopic recanalization site was visible under endoscopic ultrasonography. **b** The normal intestinal mucosa of the original endoscopic recanalization site was observed under endoscopic ultrasonography

The follow-up situation at 3 months after discharge was as follows. The patient returned to a normal diet from a fluid diet and achieved normal exhaust and defecation. The results of colonoscopy showed that the anastomotic stoma healed well, and no obvious stenosis was found. At the same time, biopsy and pathological examination were carried out on the new tissue of the original anastomotic closure with anastomotic separation. The pathological results indicated that this site was normal intestinal mucosal tissue (Fig. 4). Further endoscopic ultrasonography also confirmed the growth of normal intestinal mucosal tissues at the original anastomotic recanalization site (Fig. 5).

## Discussion and conclusions

The recovery of the anastomosis after radical colorectal cancer surgery is a long-standing concern of gastrointestinal surgeons. Clinicians need to address various anastomotic complications. The management of COA after radical resection of colon cancer is rarely reported in the literature, and the occurrence of anastomotic separation is even rarer. We presented a rare case of COA with anastomotic separation after radical resection for sigmoid colon cancer and shared our experience with the rational application of endoscopic techniques to manage such complications.

The diagnosis of COA or anastomotic separation after radical resection of colon cancer was performed by means of CT, iodine water contrast, barium enema, and endoscopy [4, 5]. Some acute patients discover the occurrence of complete anastomotic closure when symptoms, such as pain, bleeding, and others, occur after surgery. Most chronic patients have no obvious symptoms or discomfort, and thus, the condition needs to be detected by using the abovementioned inspection methods. Abdominal CT, as a common follow-up examination method after radical resection of gastrointestinal cancer [6–9] helps indicate the existence of complete anastomotic closure or anastomotic separation. Iodine water radiography and barium enema are important auxiliary examination means to evaluate the anastomotic situation of gastroenteric surgery [10–13]. They play a very important role in the diagnosis of complete anastomotic closure and anastomotic separation, in the evaluation of the degree of anastomotic separation, and in the expansion of both sides of the intestinal tube. In the present case, the separation distance of the anastomotic stoma was estimated to be about 3 cm by means of barium enema, and no contrast extravasation or fistula formation was observed in the well-dilated intestines, thereby providing the basis for our attempt to use endoscopy. Endoscopic examination can be used as one of the diagnostic criteria for completely occluded anastomosis and anastomotic separation. It can intuitively evaluate the degree of anastomotic separation, as well as the growth of both sides of the intestine and blood supply. It can provide a basis for clinicians to decide whether to perform surgery or endoscopic surgery. In this case, endoscopy was performed from the anal and ileostomy sides to evaluate the situation of anastomotic separation under endoscopic vision, as well as the softness and blood supply of the intestinal tubes on both sides. Thus, we decided to explore and solve the actual tissue structure changes in complete anastomotic closure with anastomotic separation through endoscopic incision.

No definitive conclusion was drawn on the treatment plan of postoperative COA with separation. However, considering that intestinal separation may cause the risk of abdominal infection and bleeding and that COA interferes with the structural reconstruction and functional recovery of the digestive tract [14], surgery is the routine first choice when treating this condition. However, with the improvement of endoscopic techniques [15–17], the diversification of operations, and mature application of such techniques in the management of anastomotic complications [1], surgery is not the absolute solution for postoperative complications of digestive tract. At present, endoscopic operation has achieved good clinical efficacy in many gastrointestinal anastomotic complications, such as anastomotic bleeding [18, 19], anastomotic leakage [20–22], anastomotic stenosis [23, 24], anastomotic obstruction, and others. By searching for relevant studies on severe anastomotic stenosis and closure after gastrointestinal surgery, we can summarize the experience of clinicians in the treatment of similar problems [25–35] (Table 1). In these studies, endoscopic techniques have successfully solved the problem of severe postoperative anastomotic stenosis or even closure in patients through diverse operations including balloon dilation, metal stent implantation, and retrograde endoscopic treatment. The advantages of high remission rate, high cure rate, low invasiveness, and few complications have been demonstrated. In cases of severe anastomotic stenosis, the stenosis is dilated stepwise by endoscopic insertion of a guidewire and a dilating balloon over the guidewire. Endoscopic incision can be selectively applied according to the specific situation to help the dilation of the stenosis. No definitive conclusion has been drawn on the need for metal stent implantation, but based on clinical experience, the application of self-expanding coated metal stents is found to be safe and effective for the relief of severe anastomotic stenosis. The timing of stent removal is determined by the patient’s specific feeding status, hematological findings, colonoscopy, and imaging findings. For complete anastomotic closure, in addition to surgical intervention, endoscopic treatment needs to consider more details, as follows: the activity, blood supply, and softness of the blind intestinal tube; the determination of the direction of endoscopic incision; and the choice of procedures, i.e., balloon dilation or metal stenting.

**Table 1:** Summary of the studies that reported on the treatment of completely occluded anastomosis

Case	Reference	Year	Country	Gender	Age	Primary surgery	Level of anastomosis	Diagnosis	Treatment
1	Bong [25]	2019	South korea	M	49	Low anterior resection	Low anastomosis	COA	Transanal minimally invasive surgery
2	Chen [26]	2018	China	M	66	End-to-end anastomosis and ileocecal stoma	Colorectal anastomosis	COA	Endoscopic incision
3	Curcio [27]	2010	Italy	M	70	Low anterior resection	Low anastomosis	COA	Non-electrosurgical endoscopic approach before balloon dilatation
4	D'Ambrosio [28]	2020	Italy	F	36	Modified Duhamel operation	Low anastomosis	COA	Transanal Endoscopic icrosurgery - Endoscopy ssisted treatment
5	De Lusong [29]	2008	USA	F	40	Sigmoidectomy	Colorectal anastomosis	COA	Using a prototype forward-array echoendoscope and facilitated by SpyGlass
6	Gornals [30]	2015	Spain	M	66	Low anterior resection	Low anastomosis	COA	Endoscopic ultrasound-guided and using a lumen-apposing metal stent
7	Moyer [31]	2017	USA	F	30	Partial transverse colectomy	Transverse colocecal anastomosis	COA	Using the combined antegrade-retrograde dilation procedure
8	Nasir [32]	2020	USA	M	44	Low anterior resection	Low anastomosis	COA	Rendezvous endoscopy and using transillumination to create a lumen in a complete anastomotic stricture
9	Nunes [33]	2019	Portugal	M	57	Colorectal anastomosis with protective ileostomy	Low anastomosis	COA	Endoscopic ultrasound-guided recanalization and using a lumen-apposing metal stent
10	Yazawa [34]	2014	Japan	M	79	Redo rectal resection	Low anastomosis	COA	Endoscopy: blunt penetration technique
11	Yuan [35]	2019	China	M	67	Low anterior resection and single barrel ileostomy	Anastomosis at 8 cm from the anal verge	COA	Incision was made by a needle knife and sequentially dilated by using a wire-guided balloon dilator

*M* male, *F* female, *COA* completely occluded anastomosis

The most unique aspect of the present case is the fact that COA combined with the separation of the anastomosis. Thus, managing the COA while reconstructing the continuity of the separated bowel through endoscopic manipulation was challenging. After careful assessment of the intestinal wall at the anastomosis by imaging and endoscopy, we performed an endoscopic hook incision using a titanium clip marker on the other blind side as a guide. All processes were performed under fluoroscopy to ensure the correct direction of advancement. The incision for COA should be made slowly layer by layer to avoid damaging important anatomical structures, especially in this case, where an area of unknown structure caused by anastomotic separation was present in the intestine. As for the treatment of anastomotic separation, we skillfully took advantage of the characteristics of self-expanding metal stents (SEMS). After the intestinal tube was expanded step by step through the dilating balloon guided by the guide wire, we implanted SEMS to expand the intestinal tube and bridged the separated intestinal tubes simultaneously. We used the stretchability of SEMS to gradually pull the separated intestine together, while the intestine gradually grew along the SEMS. Thus, the continuity of the intestinal tubes was reconstructed in a way similar to the primary wound healing. During the follow-up endoscopic examination, the pathological examination results of tissue biopsy confirmed the reasonable conjecture that normal intestinal mucosal tissue grew at the site of the original anastomotic separation.

In conclusion, endoscopic techniques can be an optional treatment when patients with colon cancer present with postoperative complications of severe anastomotic stenosis, closure, or separation. In our report, endoscopic technology can safely and effectively solve problems, such as COA and separation, which need to be explored and verified further through clinical studies.

## Data Availability

The datasets used and/or analyzed during the current study are available from the corresponding author on reasonable request.

## Abbreviations

COA: Completely occluded anastomosis
CT: Computed tomography
SEMS: Self-expand metal stent

## References

[1] Clifford RE, Fowler H, Govindarajah N, Vimalachandran D, Sutton PA (2019). Early anastomotic complications in colorectal surgery: a systematic review of techniques for endoscopic salvage. Surg Endosc.

[2] Davis B, Rivadeneira DE (2013). Complications of colorectal anastomoses: leaks, strictures, and bleeding. Surg Clin N Am.

[3] Ho YH (2006). Techniques for restoring bowel continuity and function after rectal cancer surgery. World J Gastroenterol.

[4] Albertsmeier M, Rittler P, Hoffmann RT, Spelsberg F (2011). Treatment of a completely obstructed colonic anastomotic stricture using a CT-guided endoscopic rendezvous technique. Endoscopy.

[5] Probst A, Gölder S, Knöpfle E, Axt L, Messmann H (2015). Computed tomography-guided endoscopic recanalization of a completely obstructed rectal anastomosis. Endoscopy.

[6] Daly B, Sukumar SA, Krebs TL, Wong JJ, Flowers JL (1996). Nonbiliary laparoscopic gastrointestinal surgery: role of CT in diagnosis and management of complication. AJR Am J Roentgenol.

[7] Pouli S, Kozana A, Papakitsou I, Daskalogiannaki M, Raissaki M (2020). Gastrointestinal perforation: clinical and MDCT clues for identification of aetiology. Insights Imaging.

[8] Ramos-Andrade D, Andrade L, Ruivo C, Portilha MA, Caseiro-Alves F, Curvo-Semedo L (2016). Imaging the postoperative patient: long-term complications of gastrointestinal surgery. Insights Imaging.

[9] Scardapane A, Brindicci D, Fracella MR, Angelelli G (2005). Post colon surgery complications: imaging findings. Eur J Radiol.

[10] Dolinsky D, Levine MS, Rubesin SE, Laufer I, Rombeau JL (2007). Utility of contrast enema for detecting anastomotic strictures after total proctocolectomy and ileal pouch-anal anastomosis. AJR Am J Roentgenol.

[11] Goetz A, da Silva NPB, Moser C, Agha A, Dendl LM, Stroszczynski C, Schreyer AG (2017). Clinical Value of Contrast Enema Prior to Ileostomy Closure. RoFo: Fortschritte auf dem Gebiete der Rontgenstrahlen und der Nuklearmedizin.

[12] Habib K, Gupta A, White D, Mazari FA, Wilson TR (2015). Utility of contrast enema to assess anastomotic integrity and the natural history of radiological leaks after low rectal surgery: systematic review and meta-analysis. Int J Colorectal Dis.

[13] Yang X, Lovell JF, Zhang Y (2019). Ingestible contrast agents for gastrointestinal imaging. ChemBioChem.

[14] Hanna MH, Vinci A, Pigazzi A (2015). Diverting ileostomy in colorectal surgery: when is it necessary?. Langenbecks Arch Surg.

[15] Neumann H, Bisschops R (2019). Artificial intelligence and the future of endoscopy. Digest Endosc.

[16] Rees CJ, Koo S, Oppong KW (2018). Future directions in therapeutic gastrointestinal endoscopy. Lancet Gastroenterol Hepatol.

[17] Zhang L, Gerson L, Maluf-Filho F (2018). Systematic review and meta-analysis in GI endoscopy: why do we need them? How can we read them? Should we trust them?. Gastrointest Endosc.

[18] Lee S, Ahn JY, Na S, Na HK, Jung KW, Kim DH, Lee JH, Choi KD, Song HJ, Lee GH (2017). Clinical features of postoperative anastomotic bleeding after gastrectomy and efficacy of endoscopic hemostasis: a case-control study. Surg Endosc.

[19] Lou Z, Zhang W, Yu E, Meng R, Fu C (2014). Colonoscopy is the first choice for early postoperative rectal anastomotic bleeding. World J Surg Oncol.

[20] Aryaie AH, Singer JL, Fayezizadeh M, Lash J, Marks JM (2017). Efficacy of endoscopic management of leak after foregut surgery with endoscopic covered self-expanding metal stents (SEMS). Surg Endosc.

[21] Bemelman WA, Baron TH (2018). Endoscopic management of transmural defects, including leaks, perforations, and fistulae. Gastroenterology.

[22] Rogalski P, Daniluk J, Baniukiewicz A, Wroblewski E, Dabrowski A (2015). Endoscopic management of gastrointestinal perforations, leaks and fistulas. World J Gastroenterol.

[23] Biraima M, Adamina M, Jost R, Breitenstein S, Soll C (2016). Long-term results of endoscopic balloon dilation for treatment of colorectal anastomotic stenosis. Surg Endosc.

[24] Lamazza A, Fiori E, Schillaci A, Sterpetti AV, Lezoche E (2014). Treatment of anastomotic stenosis and leakage after colorectal resection for cancer with self-expandable metal stents. Am J Surg.

[25] Bong JW, Lim SB (2019). Transanal minimally invasive surgery as a treatment option for a completely occluded anastomosis after low anterior resection: a new approach to severe anastomotic stenosis. Asian J Endosc Surg.

[26] Chen HL, Liu W, Jiang S, Ye LS, Zhang YH, Zeng HZ, Hu B (2018). A completely occluded colorectal anastomotic stenosis treated using an endoscopic incision method. Am J Gastroenterol.

[27] Curcio G, Spada M, di Francesco F, Tarantino I, Barresi L, Burgio G, Traina M (2010). Completely obstructed colorectal anastomosis: a new non-electrosurgical endoscopic approach before balloon dilatation. World J Gastroenterol.

[28] D'Ambrosio G, Lamazza A, Palma R, Picchetto A, Panetta C, Trecca A, Pontone S, Lezoche E (2020). Transanal endoscopic microsurgery—endoscopy assisted treatment of colorectal anastomotic stenosis. Ann Coloproctol.

[29] De Lusong MA, Shah JN, Soetikno R, Binmoeller KF (2008). Treatment of a completely obstructed colonic anastomotic stricture by using a prototype forward-array echoendoscope and facilitated by SpyGlass (with videos). Gastrointest Endosc.

[30] Gornals JB, Albines G, Trenti L, Mast R, Frago R (2015). EUS-guided recanalization of a complete rectal anastomotic stenosis by use of a lumen-apposing metal stent. Gastrointest Endosc.

[31] Moyer MT, Mathew A, Chintanaboina J, Williams E, Puleo F, Messaris E, Tinsley A (2017). Restoration of colonic patency of a completely obstructed Crohn's stricture using the combined antegrade-retrograde dilation procedure. VideoGIE.

[32] Nasir UM, Rodgers B, Choi C, Panchal D, Salimi Q, Ahlawat S (2020). A novel approach to dilation of complete colorectal anastomotic stricture using transillumination. Endosc Ultrasound.

[33] Nunes G, Marques PP, Patita M, Allen M, Gargaté L (2019). EUS-guided recanalization of complete colorectal anastomotic stenosis using a lumen-apposing metal stent. Endosc Ultrasound.

[34] Yazawa K, Morioka D, Matsumoto C, Miura Y, Togo S (2014). Blunt penetration technique for treatment of a completely obstructed anastomosis after rectal resection: a case report. J Med Case Rep.

[35] Yuan X, Liu W, Ye L, Wu M, Hu B (2019). Combination of endoscopic incision and balloon dilation for treatment of a completely obstructed anastomotic stenosis following colorectal resection: a case report. Medicine.

